# Thyroid papillary carcinoma with an insular component metastasizing to the sella turcica and sphenoid sinus: Case report

**DOI:** 10.1016/j.ijscr.2020.01.049

**Published:** 2020-02-06

**Authors:** H. Ardhaoui, S. Halily, Z. Elkrimi, S. Rouadi, R. Abada, M. Roubal, M. Mahtar

**Affiliations:** Department of Oto-Rhino-Laryngology, Head and Neck Surgery, Casablanca University Hospital, Casablanca, Morocco

**Keywords:** Papillary carcinoma, Insular component, Sphenoid metastasis

## Abstract

**Introduction:**

Papillary carcinoma accounts for approximately 80% of all thyroid carcinomas. It is associated with relatively good survival. Distant metastases occur in approximately 10% of the patients, with the lung and bone being the most commonly reported sites.

We present a case of unusual metastasis to the sphenoid bone and sella turcica from papillary thyroid carcinoma with an insular component.

**Case presentation:**

We present a case of 70 years old female patient who presents a voluminous goiter with an 11 cm mass of the left sixth rib. Trans-parietal biopsy proved its metastatic origin from a thyroid papillary carcinoma. The patient was treated with total thyroidectomy and radiation therapy as the metastatic tissue is radioiodine refractory. Pathology revealed a papillary carcinoma with an insular component. A year later, the patient develops another metastasis to the sphenoid bone extending to the sella turcica, cavernous sinus, and carotid arteries. Treatment was based on kinase inhibitor.

**Discussion:**

Metastatic invasion of the skull develops in 2.5%–5.8% of differentiated thyroid carcinoma and mostly affects the sella turcica, pituitary gland, cavernous sinus and sphenoid sinus.

The presence of an insular component in a well-differentiated thyroid carcinoma seems to be associated with a poor prognosis.

For cases where the metastatic disease is found to be resistant to conventional therapies, some clinical trials show promise with the use of tyrosine kinase inhibitors such as Sorafenib.

**Conclusion:**

Management of such uncommon cases remains challenging and should take in consideration evidence based guidelines, prognostic factors, disease progression path and treatment morbidity.

## Highlights

• The metastatic invasion of the skull develops in 2.5%–5.8% of the cases and mostly affects the sella turcica, pituitary gland, cavernous sinus and sphenoid sinus.
• The presence of an insular component in a well-differentiated thyroid carcinoma seems to be associated with a poor prognosis.
• Besides surgery and external beam radiation, kinase inhibitors seem to be the best option in radioiodine refractory tumor.

## 1 Introduction

Thyroid cancer is the most common endocrinal malignancy in the world, with an incidence that continues to rise worldwide in the last three decades [1]. The most common malignant tumor of the thyroid is papillary carcinoma. It accounts for approximately 80% of all thyroid carcinomas [2]. Most papillary thyroid carcinomas (PTC) are associated with relatively good survival, even in the metastatic setting [3]. However, some variants of PTC may behave more aggressively than classic PTC [3].

Distant metastases (DM) occur in approximately 10% of the patients with a PTC, with the lung and bone being the most commonly reported sites [1].

We present a case of unusual metastasis to the sphenoid bone and sella turcica from PTC with an insular component. The work has been reported in line with the SCARE criteria [11].

## 2 Case report

**Fig. 1:**
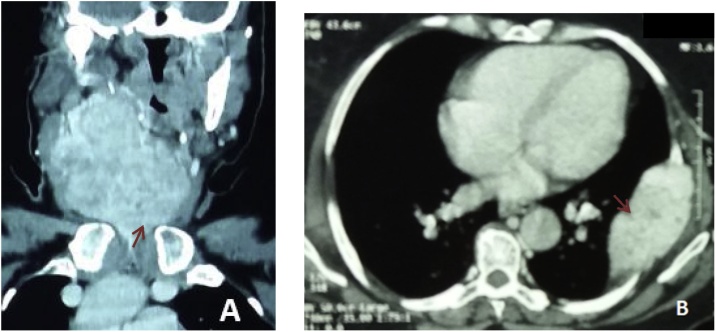
CT images showing the voluminous plunging goiter (A) and its costal metastasis (B).

**Fig. 2:**
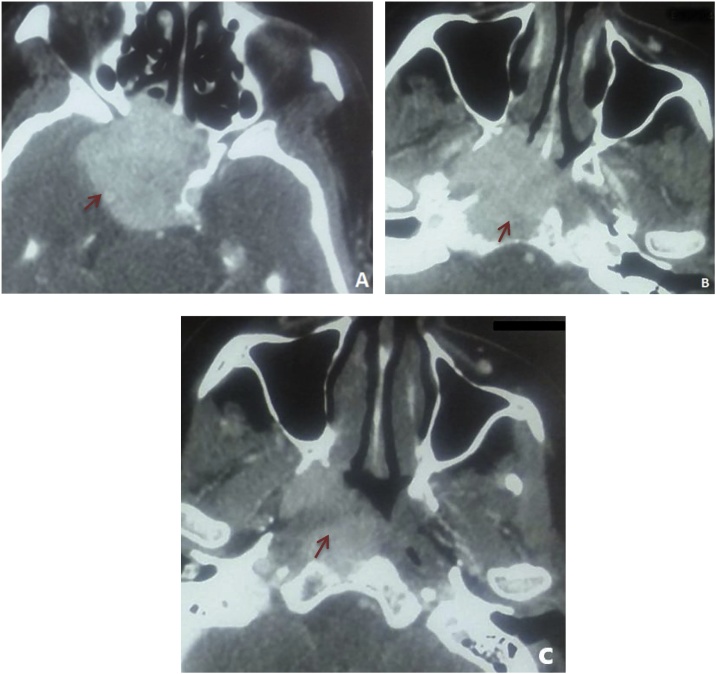
CT scan images showing the tumor of the sella turcica, extending to the sphenoid sinus (A), the nasopharynx (B) and the para-pharyngeal space (C).

**Fig. 3:**
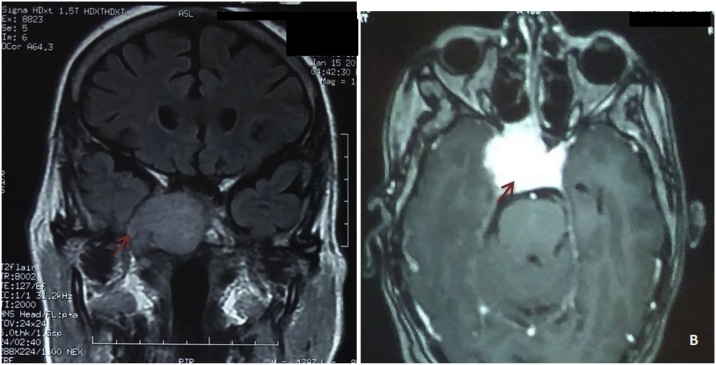
Frontal (A) and axial (B) T1-weighted MRI with gadolinium showing the tumor of the sella turcica.

We present a case of 70 years old female patient with no medical history who presented to the otolaryngology clinic with a large cervical mass evolving since two years. She also experienced dysphonia and dyspnea on exertion. Neck and chest computed tomography (CT) scans revealed a voluminous plunging goiter measuring 11 cm with jugular thrombus and an 11 cm mass of the left sixth rib (Fig. 1). Trans parietal biopsy of this mass proved its metastatic origin from a PTC. CT scans did not show other metastasis. The patient was treated with total thyroidectomy. On surgical exploration, the tumor extended to the larynx, the right recurrent laryngeal nerve and right internal jugular vein which was ligated. Tumor resection was not R0 as some tissue was left over the larynx because we did not have consent for surgery on the larynx. Final pathology revealed a multifocal papillary carcinoma with poorly differentiated focal insular component (30%), thyroid capsule rupture and vascular emboli. Radioiodine therapy was not indicated in this patient as metastatic tissue is refractory to radioiodine. She underwent adjuvant radiation therapy and was kept on TSH-suppressive thyroid hormone therapy. A year later, the patient develops mild chronic headaches with right eye ptosis without decrease in visual acuity. Head CT scans and MRI showed a tumor in the sella turcica measuring 38 × 33 mm, extending to the cavernous sinus, sphenoid sinus, nasophrynx, para-pharyngeal and pre vertebral spaces. The mass was compressing the right internal carotid artery and the optic chiasm (Fig. 2, Fig. 3).

Biopsy of the mass was performed under general anesthesia, via an endoscopic endonasal approach. Pathology confirmed metastasis from PTC. The patient was treated with a kinase inhibitor (Sorafenib). In her six month follow up, Ultrasound revealed local recurrence, sphenoid bone metastasis size slightly increased, came into contact with optic nerve which caused a decrease in visual acuity.

## 3 Discussion

Differentiated thyroid cancer (DTC), which includes papillary and follicular cancer, comprises the vast majority (>90%) of all thyroid cancers [4].

Of the differentiated cancers, papillary cancer comprises about 85% of cases compared to about 12% that have follicular histology, including conventional and oncocytic (Hürthle cell) carcinomas, and <3% that are poorly differentiated tumors [5].

Only 2%–13% of the patients affected with DTC develop bony metastasis. Therefore, this decreases the survival rate by more than 60%. The axial skeleton is the primary site of metastatic spread, with the most common sites of metastases being the spine (34.6%), the pelvis (25.5%), sternum and ribs (18.3%), extremities (10.2%), shoulder girdle (5.4%), and the cranio-maxillofacial bones (5.4%). The metastatic invasion of the skull develops in 2.5%–5.8% of the cases and mostly affects the sella turcica, pituitary gland, cavernous sinus and sphenoid sinus [1,6].

Our patient falls into the rare category of bone metastasis to the rib and sphenoid bone extending to the sella turcica and cavernous sinus which worsens prognosis and lowers chances of remission.

In addition to the poor prognostic factors already known in well differentiated carcinoma (elevated age at initial diagnosis, the presence of DM at presentation, extra thyroidal extension, vascular invasion and large tumor size), histologic factors have also been implicated. The presence of an insular component in a well DTC seems to be associated with a worse prognosis. This tumor subtype exhibits clinical and morphologic characteristics that are intermediate to those of well DTC and undifferentiated (anaplastic) thyroid carcinoma; as such, it is classified among poorly differentiated thyroid cancers [7].

There is no proven correlation between proportion of poorly differentiated carcinoma areas in the tumor and prognosis. Studies have reported similarly decreased survival in patients with poorly differentiated carcinoma constituting more than 50% of the tumor and in those with a minor component [5,8].

In a review of 111 cases of thyroid carcinoma with DM, Decaussin et al. highlight the importance of a poorly differentiated component (insular or equivalent) within a well DTC. They demonstrated that this histologic variant could predict a poor evolution. Thus, treatment of this subset of patients could be different using more aggressive therapies [8].

The conventional approach for management of a metastatic PTC includes a total thyroidectomy, along with the removal of any resectable metastatic lesions followed by radioactive iodine (RAI) and/or external beam radiation at the sites of metastases [1].

In this case report, the patient presented all poor prognostic factors mentioned above. Therefore, we preferred radiation therapy to the bony metastasis of the rib instead of surgery as we considered surgical morbidity to be high especially the large defect it would cause (11 cm mass) and its impact on respiratory function in an older patient with a deteriorated state.

Otherwise, RAI therapy was not an option because metastases were refractory to radioiodine as there was no ^131^I uptake in metastatic tissue and because of the risk of cerebral herniation secondary to edema.

According to the guidelines for radioiodine therapy of DTC, the presence of neurological symptoms or damage is a relative contraindication to RAI therapy as inflammation and local edema caused by the RAI therapy of the metastases could generate severe compression effects [9].

For cases where the metastatic disease is found to be resistant to conventional therapies, some clinical trials show promise with the use of anti-angiogenic tyrosine kinase inhibitors such as Sorafenib [5].

According to the2015 American thyroid association guidelines, kinase inhibitor therapy should be considered in RAI refractory DTC patients with metastatic, rapidly progressive, symptomatic, and/or imminently threatening disease not otherwise amenable to local control using other approaches [5].

Sorafenib was approved for use in the United States and the European Union for patients with advanced RAI-refractory DTC on the basis of a randomized placebo-controlled clinical trial (phase 3) demonstrating delayed time to disease progression among kinase inhibitor treated patients relative to those treated with a placebo [10].

Regarding cytotoxic chemotherapy, there is not enough evidence to recommend a specific regimen. It is mainly considered in RAI refractory DTC patients with metastatic, rapidly progressive, symptomatic, and/or imminently threatening disease not otherwise amenable to control through other approaches, including kinase inhibitors [5].

## 4 Conclusion

This patient presents the problem of metastatic RAI refractory DTC with an insular component. This latter is probably responsible for the aggressiveness and rapid extension of the tumor. Sphenoid bone metastases of thyroid carcinoma are uncommon and pose the problem of local extension to noble structures such as cavernous sinus, optic nerve and carotid artery.

Besides surgery and external beam radiation, kinase inhibitors seem to be the best option in RAI refractory tumor. They were well tolerated for this patient with sufficient data from the literature of delayed disease progression [10]. Otherwise, cytotoxic chemotherapy does not seem suitable in this case as it will increase morbidity without sufficient proof of efficacy.

Management of such uncommon cases remains challenging and should take in consideration evidence based guidelines, prognostic factors, disease progression path and treatment morbidity.

## Declaration of Competing Interest

None.

## Sources of funding

None.

## Ethical approval

The study is exempt from ethical approval in our institution as it is a “Case report” and not a research study.

## Consent

Written informed consent was obtained from the patient for publication of this case report and accompanying images. A copy of the written consent is available for review by the Editor-in-Chief of this journal on request.

## Registration of research studies

This is a Case report that does not require a research registry.

## Guarantor

H. Ardhaoui, S. Halily.

## Provenance and peer review

Not commissioned, externally peer-reviewed.

## CRediT authorship contribution statement

**H. Ardhaoui:** Investigation, Resources, Writing - original draft, Writing - review & editing, Visualization. **S. Halily:** Investigation, Resources, Writing - review & editing. **Z. Elkrimi:** Writing - review & editing. **S. Rouadi:** Validation, Supervision. **R. Abada:** Validation, Supervision. **M. Roubal:** Validation, Supervision. **M. Mahtar:** Validation, Supervision.
